# Supplementary material for: Global ocean redox changes before and during the Toarcian Oceanic Anoxic Event
Source: Nat Commun. 2023 Feb 13;14:815. doi: 10.1038/s41467-023-36516-x (PMC9925726; doi:10.1038/s41467-023-36516-x)
Supplement: Supplementary file 1 — Supplementary Information [file 41467_2023_36516_MOESM1_ESM.pdf]

Supplementary Information for:

Global ocean redox changes before and during the Toarcian Oceanic Anoxic Event

Alexandra Kunert and Brian Kendall

Age Correlation to Other Canadian Sections

Placement of the negative carbon isotope excursion (N-CIE) in a chronostratigraphic context is essential to validate the interpretations made here and in the main text. Foremost, it is necessary to verify that the N-CIE is undoubtedly that of the Toarcian Oceanic Anoxic Event (T-OAE) and not an unrelated excursion. Both radiometric dates and biostratigraphic methods have been interpreted from core and outcrop locations of the Gordondale Member and equivalent strata within the Western Canada Sedimentary Basin (WCSB). Here, a review of those localities with valid age information and with carbon isotope data is undertaken to correlate our carbon isotope profile with other sections and deduce a relative age constraint.

Many localities feature biostratigraphic ammonite ages when specimens are discovered during investigation. Two cores (6-32-78-5W6 and 1-35-62-20W5) and one outcrop (East Tributary, Bighorn Creek) feature carbon isotope profiles[1,2] and ammonite biostratigraphy[1,3] and are presented in **Figure S1**. Both cores and outcrop contain early- to mid-Toarcian ammonites *Harpoceras* and *Orthodactylites*, and an unidentified *Dactylioceratid* species within the N-CIE interval[1,3]. No ammonite fossils were detected in the study core (c-B6-A 94-B8).

Radiometric techniques include Re–Os black shale dates in cores 6-32-78-5W6, 7-31-79-10W6, and 13-28-73-21W5[4] and U–Pb bentonite ages from the East Tributary outcrop section[2]. The oldest Re–Os age was Sinemurian (193.83 ± 1.34 Ma), measured in the 13-28-73-21W5 core above the unconformable contact with underlying Triassic strata [4]. This Sinemurian date is difficult to directly correlate to our core as the contact between the Gordondale Member and the underlying unit is a diachronous unconformity surface throughout the WCSB. In northeastern British Columbia where our core is located, the Gordondale Member overlies Late Triassic strata, but farther east in Alberta the Early Jurassic overlies progressively older strata. Thus, we do not attempt to correlate the 193.83 Ma date to our core. In the 6-32-78-5W6 core, an age of 191.98 ± 3.88 Ma was reported within strata hosting interbedded bentonite layers[4] similar to intervals observed in the studied core. This core also features $\delta^{13}C$ data showing the N-CIE above these interbedded bentonite layers. Two younger ages of 183.50 ± 2.91 Ma (13-28-73-

21W5) and 180.10 ±2.68 Ma (7-31-79-10W6) were reported from just above the presumed Poker Chip Shale contact and from several meters above this contact, respectively [4].

The two U–Pb bentonite ages were presented for the East Tributary section of Bighorn Creek on the Gordondale-equivalent Red Deer Member. Both bentonites are situated below the N-CIE interval and gave Pliensbachian ages of 185.49 ± 0.16 Ma and 188.58 ± 0.17 Ma[2]. Our core, and the two cores with ammonite ages, also feature bentonite layers within a confined interval below the N-CIE which have been interpreted to represent the same ~3+ Myr interval of enhanced volcanism near the WCSB during the Early Jurassic.

The contact between the Gordondale and Poker Chip Shale members is widespread and easily recognizable on Gamma Ray profiles where values (in American Petroleum Units, API) drop from > 150 API in the Gordondale Member to < 150 API in the Poker Chip Shale. We also observe a transition from calcareous to non-calcareous compositions at this contact (from calcium content, not shown). It is through correlation of the Gamma Ray profiles from 13-28-73-21W5 and the study core c-B6-A/94-B-8 that an Early Toarcian age of ~183.50 Ma is assigned to the uppermost portion of the N-CIE in our core.

Taken together, the Re–Os, U–Pb and ammonite ages, plus the correlation of the gamma ray profiles, carbon isotope profiles and bentonite occurrences provide evidence that the Gordondale Member in our section was likely deposited between ≥ 193 Ma and ~180 Ma. The N-CIE at 1595 to 1588 metres depth (c-B6-A/94-B-8) may be associated with a Sinemurian or Pliensbachian event.

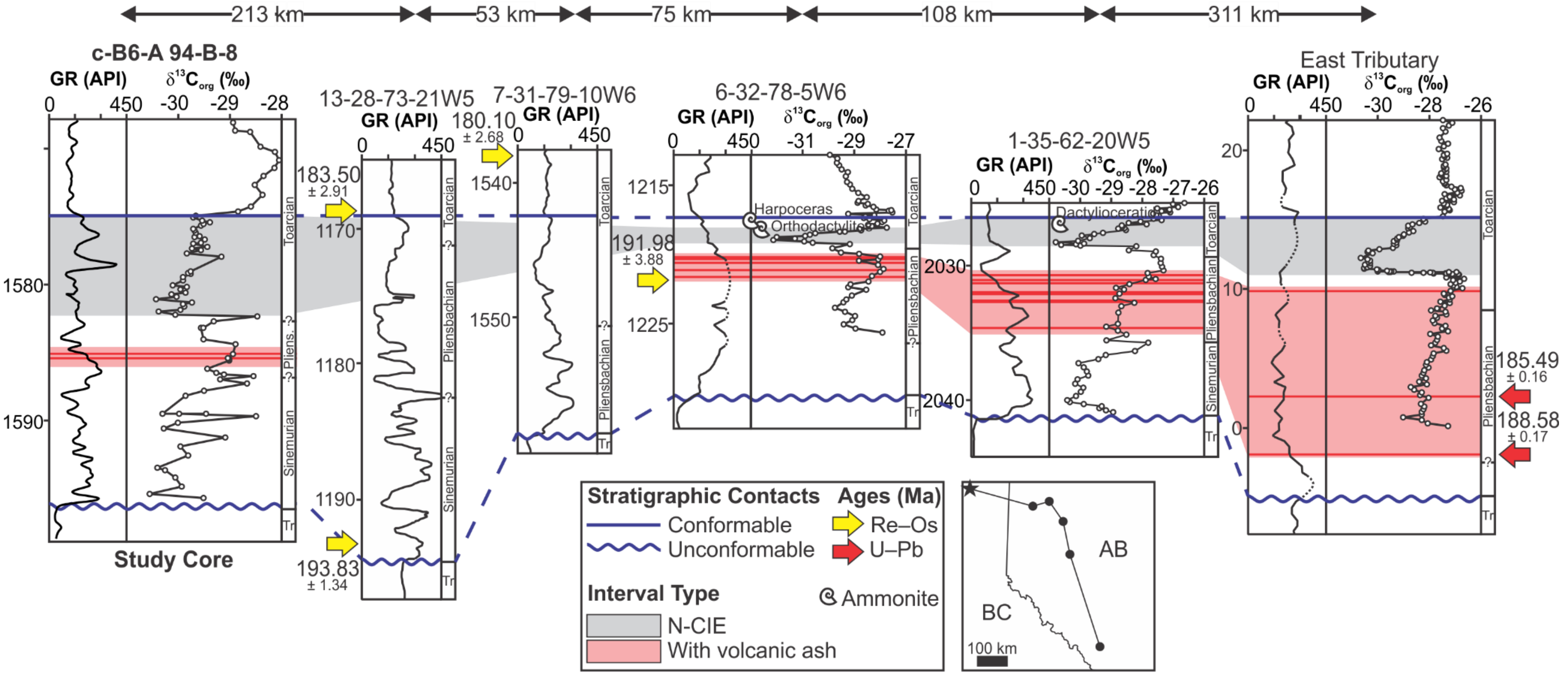

Figure S1. Age correlations between the study core (c-B6-A 94-B-8) and other Early Jurassic sections in the Western Canadian Sedimentary Basin. Panels show gamma ray (GR) in American Petroleum Institute (API) units and organic carbon isotope compositions ($\delta^{13}C_{org}$) in per mil (‰) against the international reference standard Vienna PeeDee Belemnite. Grey N-CIE (negative carbon isotope excursion) is the classical representation of the Toarcian Oceanic Anoxic Event. AB = Alberta, Canada, BC = British Columbia, Canada, Tr = Triassic, Ma = million years ago, Re–Os = rhenium–osmium age, U–Pb = uranium–lead age.

Suitability of the Gordondale Member for Global Mass Balance Modelling

The application of global trace metal mass balance models hinges on the use of black shales deposited under anoxic bottom waters that were hydrographically well-connected to global ocean circulation. Under these conditions, the sedimentary enrichment of a given redox-sensitive trace metal (e.g., Re, Mo) reflects the global ocean redox state and is used to make first-order approximations of the seafloor area covered by anoxic (Re) and euxinic (Mo) bottom waters. We discuss the redox state of the Early Jurassic Gordondale Member inferred from trace metal enrichments in the main text (see main text **Figure 2**).

To assess Gordondale Member paleohydrography, we employ Mo–U[5] and Cd/Mo versus Co*Mn[6] covariation diagrams. Both covariation techniques attempt to distinguish open-ocean conditions from restricted environments. Differences between the two techniques arise from distinctive enrichment mechanisms for U versus Cd and thus how the enrichments of these metals relate to those of Mo. It is suggested that Mo–U covariation in organic-rich sediments is controlled by three main processes[5]:

(1) Redox variation along highly productive open-ocean continental margins, resulting in a range of Mo/U where oxygenated bottom waters produce lower ratios (< modern seawater $Mo/U_{(aq)}$), and anoxic and euxinic bottom waters produce higher ratios (> modern seawater $Mo/U_{(aq)}$).

(2) Significant basin restriction resulting in initially enhanced drawdown of Mo to sediments relative to U, and development of strongly euxinic conditions depleting Mo from the water column, resulting in depressed Mo/U over time (to < 0.3× modern seawater $Mo/U_{(aq)}$).

(3) Mo adsorption to Fe-/Mn-(oxyhydr)oxide particulates in the upper water column is more efficient than U adsorption, producing enhanced local Mo concentrations in areas where particulates reach reducing bottom waters and sediments, resulting in elevated Mo/U (≥ 3× modern seawater $Mo/U_{(aq)}$).

The trends aligning with these processes were determined from modern sediment trace metal data in (1) the California, Mexico, and Peru margins[7–9]; (2) the Black Sea[10–12]; and (3) the Cariaco and Orca Basins[13].

The Cd/Mo versus Co*Mn covariation diagram is used to delineate end-member environments controlled by productivity/preservation (Cd/Mo) and open/restricted hydrography (Co*Mn)[6]:

(1) Highly productive open-ocean continental margins dominated by upwelling currents resulting in enhanced Cd deposition with organic matter relative to Mo (Cd/Mo > 0.1 approaching mean plankton Cd/Mo = 6.0)[14] and limited enrichment of Co and Mn due to depletion of these metals in upwelling waters (Co*Mn < 0.4).
(2) Weakly to strongly restricted basins with euxinic bottom waters and lower rates of primary productivity resulting in depressed Cd and thus a Cd/Mo < 0.1 (approaching seawater Cd/Mo = 0.007)[15] and enhanced Co*Mn from riverine resupply rather than Co- and Mn-depleted upwelling waters.

These trends are based on modern sediment trace metal data from (1) the Namibian, Peruvian and California Margins, and Arabian Sea[16–19], and (2) the Cariaco Basin, Saanich Inlet, and Mediterranean and Black Seas[18,20–25].

Data from the Gordondale Member is superimposed on the covariation diagrams with modern sediment trends to assess the hydrographic regime of the depositional environment (**Figure S2**). Most Gordondale Member samples plot in trend (1) for both the Mo–U and Cd/Mo versus Co*Mn covariation diagrams. Thus, both covariation diagrams suggest that the Gordondale Member was deposited in a productive, relatively open marine environment connected to global ocean circulation and receiving deep upwelling currents.

The euxinic samples[26] (square symbols) trend along the 3× seawater Mo/U line which can indicate Fe-/Mn-particulate shuttling activity[5]. However, the particulate shuttle trend from modern Cariaco Basin data occurs at Mo_{EF} and U_{EF} of ~100 and < 10, respectively. In the Gordondale Member, both Mo_{EF} and U_{EF} are elevated (~1000 and ~100, respectively), thus if a particulate shuttle was active and enhancing Mo content relative to U in reducing bottom waters then the effect was eclipsed by an overall redox control on trace metal enrichment. Regardless, Re does not have an affinity for Fe-Mn particulates and thus a particulate shuttle would have minimal impact on Re enrichments in the Gordondale Member.

The Cd/Mo versus Co*Mn covariation confirms that both anoxic and euxinic Gordondale Member samples were likely deposited under similar minor-to-negligible basin restriction conditions by Cd/Mo ratios mainly above 0.1, and Co*Mn mainly below 0.4. Based on the open-

ocean environment indicated by the covariations, the Gordondale Member from the study core was determined to be an eligible candidate for global mass balance modelling.

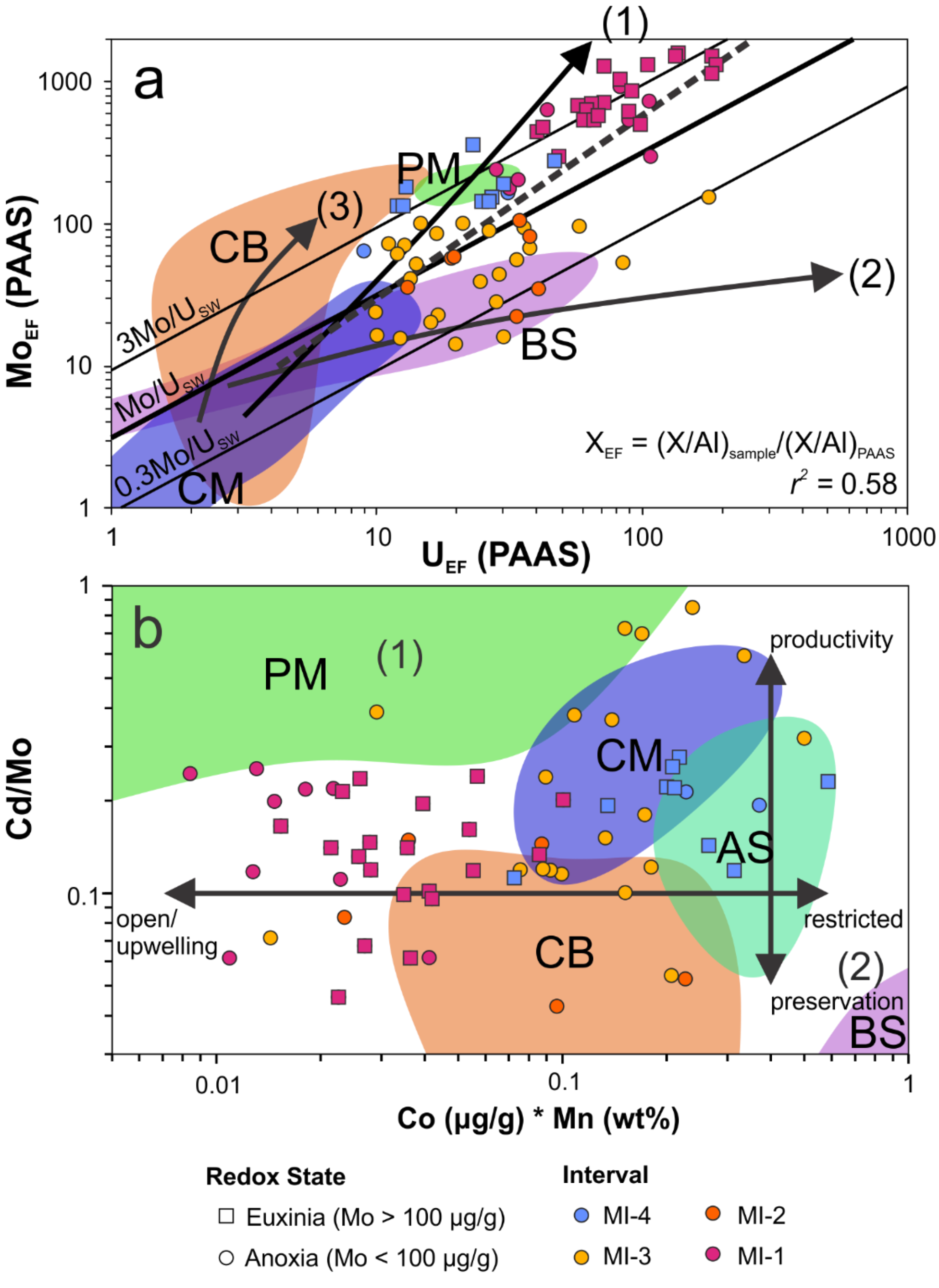

Figure S2. Paleohydrographic trends for Gordondale Member core sample data. Data points are labeled by redox state[26] and core interval. Dashed sample trends are extended for visibility. (A) Mo–U covariation diagram[5]. Enrichment factors (EF) of trace metals with respect to post-Archean average Australian shale (PAAS)[27] are used. Seawater (SW)[15], 3×SW and 0.3×SW Mo/U ratios are indicated. Trends are labeled with hydrographic controls: (1) open ocean; (2) restricted basin; and (3) particulate enhanced Mo (see text for details). Most Gordondale samples plot along the open-ocean trend. (B) Cd/Mo versus Co*Mn covariation diagram[6] with empirical Cd/Mo = 0.1 distinguishing productivity versus preservation control on enrichments, and Co*Mn = 0.4 distinguishing open versus restricted basin environments. Trends are labeled as (1) open-ocean environments in upwelling zones with high productivity and (2) restricted anoxic basins where organic matter preservation is favoured. Most Gordondale

Member samples plot in the open ocean + productivity-controlled field. Modern basins fields are Arabian Sea (AS), Black Sea (BS), California Margin (CM), Cariaco Basin (CB), and Peruvian Margin (PM).

System of Equations for Mass Balance Model

A system of equations to model anoxic seafloor area (A_{anoxic}) from authigenic Re enrichments (Re_{auth}) in sediments is derived in Sheen et al.[28] following steps outlined in Reinhard et al.[29] for Mo and Cr. The equations outlined below are written with respect to the Re model but can be applied equally to the Mo (or other trace metal) model with parameters set for the metal of interest. Three sink terms are incorporated into both Re and Mo models: oxic, dysoxic/suboxidizing (Re) or reducing (non-euxinic; Mo), and anoxic (Re) or euxinic (Mo). In the following equations, the term "intermediate" is used to represent the dysoxic/suboxidizing sink with respect to Re. For Mo this represents the reducing, but non-euxinic sink. Authigenic enrichments of trace metals are calculated by **Equation S1**.

$$\textbf{(S1)} \quad \mathrm{X_{auth}} = \mathrm{X_{sample}} - \left(\frac{\mathrm{X}}{\mathrm{Al}}\right)_{\mathrm{UCC}} \times \mathrm{Al_{sample}}$$

Where X is the metal in question, Al is aluminium, and UCC is the X and Al in the upper continental crust[30].

To facilitate modeling Re_{auth}, we assume that the intermediate seafloor area ($A_{intermediate}$) remains constant (**Table S1**), as it is not possible to deduce if the area of suboxia expanded or contracted during the T-OAE. An area of authigenically neutral seafloor ($A_{neutral}$), where the burial rate of Re to sediments ($b_{neutral}$) is 0, is present in the modern ocean (~11% of seafloor for Re, ~14% for Mo). We do not include this term in the model, instead we include this with A_{oxic} given the low burial rate of these metals in the oxic environment (3–4 orders of magnitude lower than intermediate or anoxic/euxinic environments). Given the uncertainties inherent to the model, removing the authigenically neutral 'sink' from consideration does not alter the results. The area of oxic seafloor (A_{oxic}) is calculated as the area remaining after $A_{intermediate}$ and A_{anoxic} are subtracted from the total seafloor area (**Equation S2**).

$$\textbf{(S2)} \quad A_{\mathrm{oxic}} = A_{\mathrm{total}} - A_{\mathrm{intermediate}} - A_{\mathrm{anoxic}}$$

While Re_{auth} is the value measured in black shales and used to infer A_{anoxic} for the studied time interval, the model operates in reverse to determine Re_{auth} as a function of A_{anoxic} over a range of areas from modern A_{anoxic} (~0.11% total seafloor area) to a hypothetical 100% anoxic seafloor area. The Re burial rate in anoxic sediments (b_a) is coupled to A_{anoxic} such that an

expansion of anoxic seafloor area results in a lower b_a, therefore, a new steady state anoxic burial rate (b_a') is calculated for each expansion step (**Equation S3**).

$$\textbf{(S3)} \quad b_a' = b_a^M \left(\frac{F_{in}}{\sum A_x b_x}\right)$$

Where b_a^M is the modern anoxic burial rate of Re, F_{in} is the modern riverine flux of Re into the oceans, A_x is the area of a given sink, *x* (anoxic, intermediate, oxic) and b_x is the modern burial rate of Re into a given sink, *x*. In Sheen et al.[28], F_{in} is held constant at the modern riverine input flux[31].

In addition, b_a decreases as the organic carbon burial rate (b_{Corg}) decreases with anoxic seafloor expansion towards the abyssal plain (**Equation S4**)[32], thus further reducing the burial rate of metals.

$$\textbf{(S4)} \quad b_{C_{org}}(z) = 0.5433\mathrm{e}^{-0.0009z}$$

Where *z* is water depth in metres and b_{Corg} is in mmol cm^{-2} yr^{-1}. In Sheen et al.[28], b_{Corg} is related to A_{anoxic} using a differentiable pseudo-function incorporating the bathymetric data (*z* and $A(z)$) from the eTOPO database which has a resolution of one data point per metre depth[33]. The general solution to the function is given in **Equation S5**.

$$\textbf{(S5)} \quad F_{C_{org}}(A) = \frac{1}{2}\left(b_{C_{org}}(z') + b_{C_{org}}(z)\right)(A' - A) + F_{C_{org}}(A')$$

Where $F_{Corg}(A)$ is the organic carbon flux (mmol yr^{-1}) as a function of the area of seafloor under consideration (A), A' is the area of seafloor under consideration in the previous iteration. The b_{Corg} is a function of *z* as in **Equation S4**, where *z* is the depth under consideration, and *z*' is the depth under consideration in the previous iteration. The b_a of Re at the given areal coverage of anoxia can be determined by **Equation S6**.

$$\textbf{(S6)} \quad b_a^C = \frac{F_{C_{org}}}{A_{anoxic}} A_r°(\mathrm{Re}) \cdot r$$

Where b_a^C is the C_{org}-tuned anoxic Re burial rate, A_r°(Re) is the standard atomic weight of Re, and *r* is a tunable ratio which removes b_a dependence on b_{Corg}. The b_a for a given A_{anoxic} is scaled to the b_a' and b_a^C by **Equation S7**.

$$\textbf{(S7)} \quad b_a^{scaled} = b_a' \left(\frac{b_a^M}{b_a^C}\right)$$

The final step in the system of equations is to calculate Re_{auth} from each iteration of b_a^{scaled} as a function of A_{anoxic} (**Equation S8**).

$$\textbf{(S8)} \quad \mathrm{Re_{auth}} = \frac{b_{\mathrm{a}}^{\mathrm{scaled}}}{\mathrm{BMAR}}$$

Where BMAR is the bulk mass accumulation rate. See **Table S1** for constants (modern values) for **Equations S2-S8**. For full derivation and assumptions, see Sheen et al.[28]. The same approach is taken for the Mo mass balance model using parameters from Reinhard et al.[29], and **Equations S2-S8** after Sheen et al.[28] where the intermediate sink represents reducing but non-euxinic conditions and the 'anoxic' sink is replaced with a 'euxinic' sink.

Table S1. Constants for the system of equations derived in Sheen et al.[28] with Mo parameters from Reinhard et al.[29] to determine authigenic Re and Mo concentrations as a function of anoxic seafloor area.

<table>
<tr><th>Constant</th><th>Term</th><th>Value</th><th>Model</th><th>Unit</th></tr>
<tr><td>b_a^M</td><td>Modern anoxic burial rate</td><td>1.339</td><td rowspan="3">Re</td><td rowspan="3">ng cm^{-2} yr^{-1}</td></tr>
<tr><td>b_s^M</td><td>Modern suboxic/dysoxic burial rate</td><td>0.415</td></tr>
<tr><td rowspan="2">b_o^M</td><td rowspan="2">Modern oxic burial rate</td><td>0.00160</td></tr>
<tr><td>0.00275</td><td rowspan="3">Mo</td><td rowspan="3">µg cm^{-2} yr^{-1}</td></tr>
<tr><td>b_e^M</td><td>Modern euxinic burial rate</td><td>1.53*</td></tr>
<tr><td>b_r^M</td><td>Modern reducing (non-euxinic) burial rate</td><td>0.27</td></tr>
<tr><td>$A_{suboxic}$</td><td>Area of suboxic/dysoxic seafloor</td><td>4.67</td><td rowspan="2">Re</td><td rowspan="4">% total seafloor</td></tr>
<tr><td rowspan="2">$A_{neutral}$</td><td rowspan="2">Area of authigenically neutral seafloor</td><td>11</td></tr>
<tr><td>14</td><td rowspan="2">Mo</td></tr>
<tr><td>$A_{reducing}$</td><td>Area of reducing (non-euxinic) seafloor</td><td>1.92</td></tr>
<tr><td rowspan="2">F_{in}</td><td rowspan="2">Modern input flux to ocean</td><td>429 000</td><td>Re</td><td rowspan="2">mol yr^{-1}</td></tr>
<tr><td>300 000 000</td><td>Mo</td></tr>
<tr><td>BMAR</td><td>Cariaco bulk mass accumulation rate</td><td>0.01</td><td>Re, Mo</td><td>g cm^{-2} yr^{-1}</td></tr>
<tr><td>$A_r°$(Re)</td><td>Re atomic mass</td><td>186.21</td><td>Re</td><td rowspan="2">g mol^{-1}</td></tr>
<tr><td>$A_r°$(Mo)</td><td>Mo atomic mass</td><td>95.95</td><td>Mo</td></tr>
</table>

*Excludes Black Sea values

Sensitivity Analysis

In the main text, we present the results of the Re and Mo mass balance models to estimate global anoxic and euxinic seafloor areas assuming environmentally reasonable conditions for the Early Jurassic. A sensitivity analysis was performed to ensure that our results were tested under alternative scenarios. We assess variations of BMAR, the effect of thermal maturity, sample filtering protocols, and type and magnitude of source fluxes. Thermal maturity and sample filtering affect the authigenic Re values for each interval, but not substantially enough to alter the results. The Re and Mo hydrothermal flux to the oceans is eclipsed by the riverine flux (~1.4% and ~14% of riverine, respectively), so even applying a doubling factor to the hydrothermal input does not substantially change the estimated area of global seafloor anoxia. We find that bulk mass accumulation rate and the magnitude of the riverine flux exert the greatest control on the

estimated area of anoxic seafloor. The local BMAR for a given section can be readily approximated from chronostratigraphic (section duration), thickness (section length), and bulk density parameters. However, the magnitude of riverine flux is enigmatic because accurate constraints on past continental weathering rate changes and baseline rates are difficult to obtain. We have applied the most recent estimates of the relative increase in continental weathering during the Early Jurassic[2,34]. However, our technique inherited the assumption that the intervals prior to the increase in continental weathering rate were similar to modern, and thus the magnitude of the increase is a factor of modern riverine flux.

Local Bulk Mass Accumulation Rate

The original Re mass-balance model by Sheen et al.[28] was illustrated with a Cariaco Basin BMAR of 1.0×10^{-2} g cm^{-2} yr^{-1} with factors of 1.5 above and below. While this provides an ample range for estimating seafloor anoxia, a closer approximation of the local depositional BMAR can be calculated for an interval by **Equation S9** assuming continuous sedimentation.

$$\textbf{(S9)} \quad \text{BMAR} = \frac{\rho_b \Delta z}{\Delta t}$$

Where ρ_b is mean bulk density of an interval (g cm^{-3}), Δz is vertical interval length (cm) and Δt is the interval duration (yr).

For the studied core, ρ_b of the Gordondale Member within the N-CIE interval is 2.42 ± 0.16 g cm^{-3} (1s) recorded between depths of 1575.00 m and 1582.10 m (Δz = 710 cm). Bulk densities were determined at Weatherford Labs during routine core analysis by determining sample weight (g) and using a mercury pump to determine bulk volume (cm^3). The N-CIE is estimated to have occurred over a period of 300 to 500 kyr[35–37]. Some studies suggest that the T-OAE lasted up to 1 Myr, however, this is stated as an upper estimate with the main N-CIE phase lasting a few hundred kyr[38]. Thus, we use the median Δt of 400 kyr, and minimum and maximum (300 and 500 kyr, respectively) to calculate representative median, minimum, and maximum BMAR. By **Equation S9**, the N-CIE interval median BMAR is 4.3×10^{-3} g cm^{-2} yr^{-1} within a range of 3.4–5.7 × 10^{-3} g cm^{-2} yr^{-1}. Decreasing BMAR from the Cariaco Basin to the calculated local depositional values leads to a higher estimated A_{anoxic} for a given Re_{auth} (**Table S2**). The local BMAR range determined from an event lasting 300 to 500 kyr is applied in all model calculations for the Gordondale Member data here, and only the median local BMAR (from an event lasting 400 kyr) is applied to the models in the main text.

Table S2. Comparison of authigenic Re and Mo modeling results to determine anoxic seafloor area (A_{anoxic}) using Cariaco Basin bulk mass accumulation rate (BMAR) and calculated local BMAR (bolded = median from 400 kyr event; brackets = range from 300–500 kyr event). Ranges incorporate the 1SD of the metal authigenic enrichments.

Model Interval	*n*	Re_{auth} (ng g^{-1})	A_{anoxic}		Mo_{auth} (µg g^{-1})	$A_{euxinic}$	
			Cariaco Basin BMAR	Local BMAR		Cariaco Basin BMAR	Local BMAR
4	11	277 ± 74	**0.8** (0.0–3.4)	**4.1** (1.8–8.8)	108 ± 22	**1.7** (0.7–3.7)	**4.8** (2.7–8.3)
3	25	188 ± 89	**2.0** (0.0–9.6)	**7.1** (2.7–100)	28 ± 18	Non-euxinic (Cannot Determine)	
2	7	133 ± 61	**0.1** (0.0–3.1)	**2.3** (0.4–8.0)	20 ± 11		
1	29	215 ± 88	**0.0** (0.0–1.0)	**0.8** (0.0–3.6)	168 ± 91	**0.0** (0.0–1.0)	**0.5** (0.1–2.5)

Thermal Maturity

The Gordondale Member is located within the Western Canada Sedimentary Basin which, due to the west-verging Rocky Mountain fold and thrust belt, exhibits an increasing thermal maturity gradient from northeast to southwest[39]. The cored section from this study is situated in the foothills of the Rocky Mountains in British Columbia. Thermal overmaturity with respect to hydrocarbon generation is indicated by programmed pyrolysis Tmax values greater than 470°C [40] and breakdown of the relationship between trace metals (Re_{auth} and Mo_{auth}) and TOC (**Figure S3**) through the Gordondale Member.

Thermal maturation of organic-rich rocks has not been shown to disrupt trace metal isotope systematics; however, it may cause an increase in the concentrations of trace metals due to organic mass loss[41]. With respect to the mass balance model, higher Re or Mo concentration due to overmaturity may result in an underestimation of the areal expanse of seafloor anoxia or euxinia. We attempt to model adjusted metal concentrations from the maximum organic matter content (C_{org}) of approximately 28 wt% in immature sections of the Gordondale Member[42]. The Gordondale Member in our overmature section reaches a maximum of approximately 12 wt% C_{org}, suggesting that 43% of the organic mass remains after thermal maturation of this black shale. A simple calculation to estimate pre-maturation metal concentrations (X_{pre}) is made by **Equation S10** using a representative sample mass of 1 g (because metal and C_{org} concentrations are reported per-gram).

$$\textbf{(S10)} \quad X_{pre} = \frac{X \cdot 1g}{1g + \left(\frac{C_{org} \cdot 1g}{0.43} - C_{org} \cdot 1g\right)}$$

Where the numerator represents the metal (X) in 1 g mass from the bulk overmature sample (assuming no metal loss during maturation), and the denominator represents 1 g of overmature sample mass plus the C_{org} estimated to have been lost during the maturation process. The values

of X and C_{org} are the concentrations of each component measured in the sample in ng g^{-1} for Re, μg g^{-1} for Mo, and wt% for C_{org} (10^{-2} g g^{-1}).

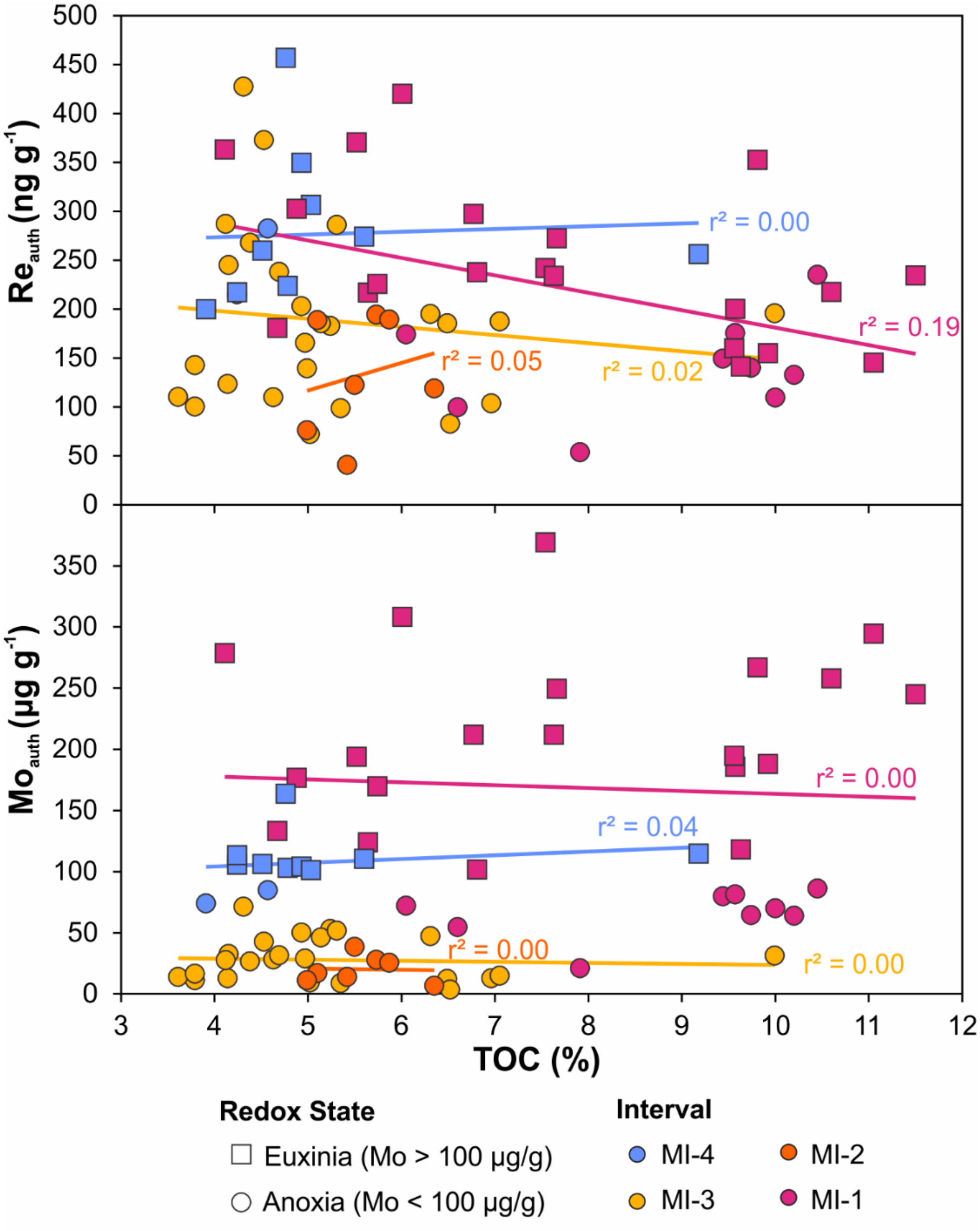

Figure S3. Covariation of authigenic rhenium (Re_{auth}) and molybdenum (Mo_{auth}) against total organic carbon (TOC) content in the Gordondale Member. No systematic relationship is observed between the trace metals and TOC which may indicate disruption or loss of organic matter during thermal maturation[43].

The correction for organic mass loss by **Equation S10** was made for each sample, and new means for Re (Re_{pre}) and Mo (Mo_{pre}) were calculated for Model Interval (MI, see Figure 2a in main text for relative positions in the core) 1 and MI-2, MI-3 and MI-4 intervals and applied to the mass balance models (**Table S3**). Overall, the adjustment decreased the total sample mean Re and Mo concentrations corresponding to anoxic and euxinic seafloor areas only slightly

greater (by < 1% global seafloor area) than those from non-adjusted concentrations. Thus, we consider the non-corrected value presented in the main text to be representative of the depositional environment regardless of section maturity.

Table S3. Sample count (*n*), mean authigenic Re and Mo concentration (± 1 standard deviation; ng g^{-1}) and mass balance model results for anoxic and euxinic seafloor area as % total seafloor (range in brackets) from measured and maturity-adjusted values (by **Equation S10**). The mass balance model results follow the assumptions stated in the main text—i.e., 3× increase in riverine flux from Pre-N-CIE to N-CIE.

Model Interval	*n*	Measured		Maturity Adjusted	
		Re_{auth}	A_{anoxic}	Re_{pre}	A_{anoxic}
4	11	277 ± 74	**4.1** (1.8–8.8)	259 ± 70	**4.6** (2.0–9.9)
3	25	188 ± 89	**7.1** (2.7–100)	176 ± 85	**7.8** (2.9–100)
2	7	133 ± 61	**2.3** (0.4–8.0)	124 ± 56	**2.6** (0.6–8.7)
1	29	215 ± 88	**0.8** (0.0–3.6)	195 ± 83	**1.1** (0.0–4.3)
**Model Interval**	***n***	Mo_{auth}	$A_{euxinic}$	Mo_{pre}	$A_{euxinic}$
4	11	108 ± 22	**4.8** (1.8–8.8)	101 ± 21	**5.2** (2.9–9.0)
3	25	28 ± 18	Non-euxinic (Cannot Determine)	26 ± 17	Non-euxinic (Cannot Determine)
2	7	20 ± 11		19 ± 10	
1	29	168 ± 91	**0.8** (0.0–3.6)	152 ± 82	**0.7** (0.0–2.8)

Sample Filtering for Anoxia

The original model of Sheen et al. used culled datasets which include only shale samples exhibiting anoxic signatures using paleoredox proxy filters[28]. This method was viable for that study due to the large datasets (*n* > 3000) covering an extensive period (>2.5 Gyr) from a variety of depositional environments. Their filtering protocols aimed to eliminate the variation inherent to this type of dataset. Our study, however, focuses on the ~10 Myr period covered by the Gordondale Member, and more specifically the < 1 Myr period leading up to and during the N-CIE of the T-OAE within a single drill core. In the main text, we did not limit our samples to those filtered using the original methods[28] because this reduces the statistical robustness of the sample set, and we find that filtering the samples does not greatly affect the results of the model as demonstrated below. Instead, we used trace metal redox proxies to determine the probable depositional conditions, and to justify the removal of 9 samples between MI-2 and MI-3 that were likely deposited under more oxygenated conditions.

Here, we show the results from two filtered datasets (**Table S4**): (1) filtered using original methods[28], and (2) filtered as shown in the main text. Filtered dataset 1 includes:

- **C_{org} > 0.4 wt%** to identify marine, fine-grained siliciclastic organic-rich mudrocks
- **Fe_T/Al > 0.5** to identify samples deposited under anoxic conditions
- **Re > 5.0 ng g^{-1}** to identify samples where the sediment column was anoxic

- Other **trace metal enrichments** (e.g., Mo) when the above were unavailable

For filtered dataset 1 with original protocols[28], we do not apply other trace metal enrichment filters as our dataset includes C_{org}, Fe_T/Al and Re. Filtered dataset 2 does not include the C_{org}, Fe_T/Al or Re filters as all samples are marine, fine-grained, organic-rich mudrocks (C_{org} > 3.6 wt%), and all samples have Re > 41 ng g^{-1}. Instead, we focus on other trace metal proxies to identify the local depositional redox conditions, which are outlined in the main text.

Filtered dataset 1 is reduced to n = 20 when strictly adhering to the original protocol[28]. This culling is due entirely to the Fe_T/Al filter (we note that if local detrital Fe_T/Al ratios were < 0.5, then there was unnecessary culling of the dataset), as all samples contain C_{org} > 0.4 wt% and Re > 5.0 ng g^{-1}. There is a decreased sample count for filtered dataset 2, but only within MI-2 (removed 2 samples) and MI-3 (removed 7 samples) due to interpreted dysoxic conditions.

Table S4. Sample count (*n*), mean authigenic Re concentration (± 1 standard deviation; ng g^{-1}) and mass balance model results for anoxic seafloor area (% total seafloor) (1) dataset filtered using original protocols[28], and (2) dataset filtered using alternative trace metal enrichment filtering. The mass balance model results shown in this table follow the assumptions stated in the main text—i.e., 3× increase in riverine flux from MI-2 to MI-3. Italicised values indicate poor statistical control (*n* = 1). Unfiltered data are also presented to show changes.

**Model Interval**	**Filtered (1)**			**Filtered (2)**			**Unfiltered**		
	n	**Re_{auth}**	**A_{anoxic}**	***n***	**Re_{auth}**	**A_{anoxic}**	***n***	**Re_{auth}**	**A_{anoxic}**
4	2	303 ± 66	**3.6** (1.6–7.0)	11	277 ± 74	**4.1** (1.8–8.8)	11	277 ± 74	**4.1** (1.8–8.8)
3	5	262 ± 76	**4.5** (1.9–10)	18	192 ± 95	**7.1** (2.7–100)	25	188 ± 89	**7.1** (2.7–100)
2	*1*	*76*	***5.4** (3.6–7.4)*	5	125 ± 68	**2.3** (0.4–8.0)	7	133 ± 61	**2.4** (0.4–8.1)
1	12	252 ± 89	**0.5** (0.0–2.4)	29	215 ± 88	**0.8** (0.0–3.6)	29	215 ± 88	**0.8** (0.0–3.6)

We observe an increased mean Re_{auth} in the filtered datasets within the uncertainty of unfiltered Re_{auth} ranges. This trend was expected due to the elimination of samples which may be non-anoxic that record lower Re enrichments. The only anomaly to that statement occurs in filtered dataset 1 in MI-2 which lies below the value of the unfiltered data (italicised values in **Table S4**). This can be overlooked as the filtered sample interval contains a single sample point, which is less statistically representative than multiple samples. We also observe that the relative enrichments of each interval are consistent regardless of the dataset, i.e., the MI-2 interval records the lowest Re_{auth} while MI-4 records the highest. A similar exercise could be undertaken with Mo_{auth}, yielding similar results.

Hydrothermal Flux

In the modern oceans, high temperature hydrothermal activity constitutes a negligible component of the Re input flux—estimated at 0.1% of the global riverine Re input[31]. Low temperature hydrothermal activity is insufficiently characterised, so no estimate of its

contribution to ocean Re input flux has been established. High and low temperature hydrothermal Mo fluxes to the ocean are 1% and 13% of Mo riverine flux, respectively[31]. This represents a total hydrothermal flux of 14% of the riverine flux. Constraints can be placed on the low temperature hydrothermal Re flux if it is assumed that Re and Mo are added to the ocean from the high and low temperature sources in a broadly similar manner. The high temperature Re flux equivalent to 0.1% riverine flux, in addition to a potential low temperature Re flux equivalent to 1.3% of riverine flux sums to a hydrothermal Re flux equivalent to 1.4% riverine flux. Due to this minor contribution of hydrothermal Re and Mo to the ocean relative to rivers, riverine input is treated as the sole source flux (F_{in}) to the modern oceans[28,29], supplying 4.29×10^5 mol yr^{-1} and 3.00×10^8 mol yr^{-1}, respectively[31]. From these riverine fluxes, hydrothermal input is calculated at 6.01×10^3 mol Re yr^{-1} and 4.20×10^7 mol Mo yr^{-1}.

The Gordondale Member was deposited through the Early Jurassic, an age which included major tectonic change, such as the breakup of the supercontinent Pangea. Rifting along plate boundaries during this period likely enhanced hydrothermal activity along new mid-ocean ridges, e.g., opening of the Hispanic Corridor[44]. A seawater $^{87}Sr/^{86}Sr$ excursion to less radiogenic values from the Pliensbachian–Toarcian boundary to the onset of the T-OAE suggests an 8 to 86% increase in submarine hydrothermal activity[45]. This modeling attempt assumes that (1) riverine flux or (2) $^{87}Sr/^{86}Sr$ of input material remained constant through the excursion. The wide range in the model results are due to the dependence on initial high temperature hydrothermal Sr flux and the ratio of low temperature to high temperature hydrothermal Sr input.

If the Re and Mo hydrothermal flux responds similarly to the Sr flux during enhanced seafloor hydrothermal events, then the total Re and Mo hydrothermal fluxes from the modern ocean likely underestimate the hydrothermal Re input to the ocean prior to the T-OAE. If the hydrothermal components are doubled (rounded up from a maximum hydrothermal Sr flux increase of 86%[45]), then the hydrothermal Re and Mo fluxes become 2.8% and 28% of the riverine flux, respectively.

Here, we assess the Re and Mo mass balance models using three source flux scenarios (**Table S5**): (1) modern riverine only, held constant; (2) modern riverine + modern hydrothermal, held constant; and (3) constant modern riverine + 2× modern hydrothermal for MI-1 and MI-2 and constant modern riverine + modern hydrothermal for MI-3 and MI-4 based on the seawater $^{87}Sr/^{86}Sr$ excursion ending prior to the onset of the N-CIE[45].

Table S5. Sample count (*n*), mean authigenic Re and Mo concentrations (± 1 standard deviation; ng g^{-1}) and mass balance model results for anoxic seafloor area as % total seafloor (range in brackets) using varied source fluxes as follows: (1) constant modern riverine, (2) constant modern riverine + hydrothermal, and (3) constant modern riverine + 2× modern hydrothermal for Model Interval 1 and 2, constant modern riverine + hydrothermal for Model Intervals 3 and 4. The local BMAR is applied for all model attempts.

**Model Interval**	***n***	**Re_{auth}**	**A_{anoxic} (%)**		
			**(1)**	**(2)**	**(3)**
4	11	277 ± 74	**0.3** (0.0–1.6)	**0.3** (0.0–1.7)	
3	25	188 ± 89	**1.2** (0.0–5.1)	**1.2** (0.0–5.3)	
2	7	133 ± 61	**2.3** (0.4–8.0)	**2.4** (0.5–8.2)	**2.5** (0.5–8.3)
1	29	215 ± 88	**0.8** (0.0–3.6)	**0.9** (0.0–3.7)	**0.9** (0.0–3.7)
**Model Interval**	***n***	**Mo_{auth}**	**$A_{euxinic}$ (%)**		
			**(1)**	**(2)**	**(3)**
4	11	108 ± 22	**1.2** (0.5–2.2)	**1.5** (0.7–2.6)	
3	25	28 ± 18	Non-euxinic (Cannot Determine)		
2	7	20 ± 11			
1	29	168 ± 91	**0.6** (0.0–2.5)	**0.7** (0.0–3.0)	**0.9** (0.4–3.4)

We observe little or no change in resulting anoxic seafloor area even when modern hydrothermal input is doubled because it is greatly eclipsed by the riverine Re and Mo input. Therefore, we do not include hydrothermal fluxes in the model presented in the main text because the low temperature fluxes are poorly characterised, and the total hydrothermal flux is greatly exceeded by the riverine flux.

Magnitude of Riverine Flux

Several geochemical lines of evidence point to increased continental weathering rates at local and global scales during the early Toarcian[2,34,46] which corresponds to our N-CIE intervals. The magnitude of global weathering rate during the T-OAE has been estimated as a 215-530% (~3- to 6-fold) increase from pre-event levels based on $^{187}Os/^{188}Os_i$ in the Pliensbachian–Toarcian Red Deer Member and Toarcian Poker Chip Shale at East Tributary, Alberta, Canada[2]. We evaluate model scenarios with 3-, 4.5- and 6-fold increases in weathering rate, and by extension riverine flux, to assess variation in A_{anoxic} (**Table S6**). Relatively minor linear increases in the riverine flux lead to exponential increases in the estimated anoxic seafloor areas from the model given the same Re_{auth} input. This indicates that the selection of the weathering rate and/or riverine flux to the oceans is a pivotal component of the spatiotemporal tuning of the Re mass balance model.

Table S6. Sample count (*n*), mean authigenic Re concentration (± 1 standard deviation; ng g^{-1}) and mass balance model results for anoxic seafloor area as % total seafloor (range in brackets) using varied riverine source fluxes as a factor of modern riverine source flux[31]. The range of source fluxes explored here are constrained from Os-isotope evaluations and applied only to N-CIE intervals[2]. The local BMAR is applied for all model attempts.

Model Interval	*N*	Re_{auth}	A_{anoxic} (%)		
			3xRiv (N-CIE)	4.5xRiv (N-CIE)	6xRiv (N-CIE)
4	11	277 ± 74	**4.1** (1.8–8.8)	**7.3** (3.5–100)	**12** (5.3–100)
3	25	188 ± 89	**7.1** (2.7–100)	**21** (4.9–100)	**100** (7.3–100)
2	7	133 ± 61	**2.3** (0.4–8.0)		
1	29	215 ± 88	**0.8** (0.0–3.6)		

This exercise can be extended to the Mo model, with the same results. As discussed in the main text, we select the most environmentally reasonable weathering rate change (3-fold increase) across the beginning of the T-OAE (N-CIE) as we believe that the range given in Them et al.[2] for the increase in continental weathering may be a mild overestimation due to greater basin restriction at the East Tributary locality compared to our core section, as inferred from the approximately four-fold smaller mean Re concentration at East Tributary. The effect of the greater basin restriction may have been a higher local seawater $^{187}Os/^{188}Os$ because of the influence of local continental inputs. For this reason, we consider increases of continental weathering of more than 3-fold to be less probable.

Supplementary References

1. Them, T. R. *et al.* High-resolution carbon isotope records of the Toarcian Oceanic Anoxic Event (Early Jurassic) from North America and implications for the global drivers of the Toarcian carbon cycle. *Earth Planet Sci Lett* **459**, 118–126 (2017).

2. Them, T. R. *et al.* Evidence for rapid weathering response to climatic warming during the Toarcian Oceanic Anoxic Event. *Sci Rep* **7**, (2017).

3. Asgar-Deen, M., Hall, R., Craig, J. & Riediger, C. New biostratigraphic data from the Lower Jurassic Fernie Formation in the subsurface of west-central Alberta and their stratigraphic implications. *https://doi.org/10.1139/e02-096* **40**, 45–63 (2011).

4. Toma, J., Creaser, R. A. & Pană, D. I. High-precision ReOs dating of Lower Jurassic shale packages from the Western Canadian Sedimentary Basin. *Palaeogeogr Palaeoclimatol Palaeoecol* **560**, 110010 (2020).

5. Algeo, T. J. & Tribovillard, N. Environmental analysis of paleoceanographic systems based on molybdenum-uranium covariation. *Chem Geol* **268**, 211–225 (2009).

6. Sweere, T., van den Boorn, S., Dickson, A. J. & Reichart, G. J. Definition of new trace-metal proxies for the controls on organic matter enrichment in marine sediments based on Mn, Co, Mo and Cd concentrations. *Chem Geol* **441**, 235–245 (2016).

7. Irino, T. & Pedersen, T. F. Geochemical character of glacial to interglacial sediments at Site 1017, Southern California margin; minor and trace elements. in *Proceedings of the Ocean Drilling Program Scientific Results* (eds. Lyle, M., Koizumi, I., Richter, C. & Moore Jr., T. C.) 263–271 (Ocean Drilling Program, 2000).

8. Tada, R., Sato, S., Irino, T., Matsui, H. & Kennett, J. P. Millennial-scale compositional variations in late Quaternary sediments at Site 1017, Southern California. in *Proceedings of the Ocean Drilling Program Scientific Results* (eds. Lyle, M., Koizumi, I., Richter, C. & Moore Jr., T. C.) (Ocean Drilling Program, 2000).

9. McManus, J. *et al.* Molybdenum and uranium geochemistry in continental margin sediments: Paleoproxy potential. *Geochim Cosmochim Acta* **70**, 4643–4662 (2006).

10. Anderson, R. F. & Fleisher, M. Q. Uranium Precipitation in Black Sea Sediments. *Black Sea oceanography* 443–458 (1991) doi:10.1007/978-94-011-2608-3_26.

11. Barnes, C. E. & Cochran, J. K. Geochemistry of uranium in Black Sea sediments. *Deep Sea Research Part A. Oceanographic Research Papers* **38**, S1237–S1254 (1991).

12. Lyons, T. W. Comparative study of Holocene Black Sea sediments from oxic and anoxic sites of deposition: geochemical and sedimentological criteria. (1992).

13. Tribovillard, N. *et al.* Paleodepositional conditions in the Orca Basin as inferred from organic matter and trace metal contents. *Mar Geol* **254**, 62–72 (2008).

14. Brumsack, H. J. The inorganic geochemistry of Cretaceous black shales (DSDP Leg 41) in comparison to modern upwelling sediments from the Gulf of California. *Geological Society, London, Special Publications* **21**, 447–462 (1986).

15. Tribovillard, N., Algeo, T. J., Lyons, T. & Riboulleau, A. Trace metals as paleoredox and paleoproductivity proxies: An update. *Chem Geol* **232**, 12–32 (2006).

16. Brongersma-Sanders, M., Stephan, K. M., Kwee, T. G. & de Bruin, M. Distribution of minor elements in cores from the southwest Africa shelf with notes on plankton and fish mortality. *Mar Geol* **37**, 91–132 (1980).

17. van der Weijden, C. H., Reichart, G. J. & van Os, B. J. H. Sedimentary trace element records over the last 200 kyr from within and below the northern Arabian Sea oxygen minimum zone. *Mar Geol* **231**, 69–88 (2006).

18. Brumsack, H.-J. Geochemistry of recent TOC-rich sediments from the Gulf of California and the Black Sea. *Geologische Rundschau 1989 78:3* **78**, 851–882 (1989).

19. Böning, P. *et al.* Geochemistry of Peruvian near-surface sediments. *Geochim Cosmochim Acta* **68**, 4429–4451 (2004).

20. Nijenhuis, I. A., Bosch, H. J., Sinninghe Damsté, J. S., Brumsack, H. J. & de Lange, G. J. Organic matter and trace element rich sapropels and black shales: a geochemical comparison. *Earth Planet Sci Lett* **169**, 277–290 (1999).

21. Morford, J. L., Russell, A. D. & Emerson, S. Trace metal evidence for changes in the redox environment associated with the transition from terrigenous clay to diatomaceous sediment, Saanich Inlet, BC. *Mar Geol* **174**, 355–369 (2001).

22. Russell, A. D. & Morford, J. L. The behavior of redox-sensitive metals across a laminated–massive–laminated transition in Saanich Inlet, British Columbia. *Mar Geol* **174**, 341–354 (2001).

23. Arnaboldi, M. & Meyers, P. A. Trace element indicators of increased primary production and decreased water-column ventilation during deposition of latest Pliocene sapropels at five locations across the Mediterranean Sea. *Palaeogeogr Palaeoclimatol Palaeoecol* **249**, 425–443 (2007).

24. Piper, D. Z. & Dean, W. E. Trace-element deposition in the Cariaco Basin, Venezuela Shelf, under sulfate-reducing conditions: a history of the local hydrography and global climate, 20 ka to the present. *Professional Paper* 1–41 (2002) doi:10.3133/PP1670.

25. Baturin, G. N. Geochemistry of sapropel in the Black Sea. *Geochemistry International 2011 49:5* **49**, 531–535 (2011).

26. Scott, C. & Lyons, T. W. Contrasting molybdenum cycling and isotopic properties in euxinic versus non-euxinic sediments and sedimentary rocks: Refining the paleoproxies. *Chem Geol* **324–325**, 19–27 (2012).

27. Taylor, S. R. & McLennan, S. M. *The continental crust: Its composition and evolution*. (U.S. Department of Energy Office of Scientific and Technical Information, 1985).

28. Sheen, A. I. *et al.* A model for the oceanic mass balance of rhenium and implications for the extent of Proterozoic ocean anoxia. *Geochim Cosmochim Acta* **227**, 75–95 (2018).

29. Reinhard, C. T. *et al.* Proterozoic ocean redox and biogeochemical stasis. *Proceedings of the National Academy of Sciences* **110**, 5357–5362 (2013).

30. Mclennan, S. M. Relationships between the trace element composition of sedimentary rocks and upper continental crust. *Geochemistry, Geophysics, Geosystems* **2**, (2001).

31. Miller, C. A., Peucker-Ehrenbrink, B., Walker, B. D. & Marcantonio, F. Re-assessing the surface cycling of molybdenum and rhenium. *Geochim Cosmochim Acta* **75**, 7146–7179 (2011).

32. Middelburg, J. J., Soetaert, K. & Herman, P. M. J. Empirical relationships for use in global diagenetic models. *Deep Sea Research Part I: Oceanographic Research Papers* **44**, 327–344 (1997).

33. Amante, C. & Eakins, B. W. *ETOPO1 1 Arc-Minute Global Relief Model: Procedures, Data Sources and Analysis*. (2009).

34. Brazier, J. M. *et al.* Calcium isotope evidence for dramatic increase of continental weathering during the Toarcian oceanic anoxic event (Early Jurassic). *Earth Planet Sci Lett* **411**, 164–176 (2015).

35. Sell, B. *et al.* Evaluating the temporal link between the Karoo LIP and climatic–biologic events of the Toarcian Stage with high-precision U–Pb geochronology. *Earth Planet Sci Lett* **408**, 48–56 (2014).

36. Boulila, S. *et al.* Astronomical calibration of the Toarcian Stage: Implications for sequence stratigraphy and duration of the early Toarcian OAE. *Earth Planet Sci Lett* **386**, 98–111 (2014).

37. Ikeda, M. & Hori, R. S. Effects of Karoo–Ferrar volcanism and astronomical cycles on the Toarcian Oceanic Anoxic Events (Early Jurassic). *Palaeogeogr Palaeoclimatol Palaeoecol* **410**, 134–142 (2014).

38. Kemp, D. B., Coe, A. L., Cohen, A. S. & Schwark, L. Astronomical pacing of methane release in the Early Jurassic period. *Nature* **437**, 396–399 (2005).

39. Riediger, C. L. Solid bitumen reflectance and Rock-Eval Tmax as maturation indices: an example from the “Nordegg Member”, Western Canada Sedimentary Basin. *Int J Coal Geol* **22**, 295–315 (1993).

40. Peters, K. E., Walters, C. C. & Moldowan, J. M. *The Biomarker Guide, Volume 1: Biomarkers and Isotopes in the Environment and Human History*. vol. 1 (Cambridge University Press, 2007).

41. Dickson, A. J., Idiz, E., Porcelli, D. & van den Boorn, S. H. J. M. The influence of thermal maturity on the stable isotope compositions and concentrations of molybdenum, zinc and cadmium in organic-rich marine mudrocks. *Geochim Cosmochim Acta* **287**, 205–220 (2020).

42. Riediger, C. L. Lower Mesozoic hydrocarbon source rocks, Western Canada Sedimentary Basin. (1991).

43. Ardakani, O. H., Chappaz, A., Sanei, H. & Mayer, B. Effect of thermal maturity on remobilization of molybdenum in black shales. *Earth Planet Sci Lett* **449**, 311–320 (2016).

44. Porter, S. J., Selby, D., Suzuki, K. & Gröcke, D. Opening of a trans-Pangaean marine corridor during the Early Jurassic: Insights from osmium isotopes across the Sinemurian–Pliensbachian GSSP, Robin Hood’s Bay, UK. *Palaeogeogr Palaeoclimatol Palaeoecol* **375**, 50–58 (2013).

45. Jones, C. E. & Jenkyns, H. C. Seawater Strontium Isotopes, Oceanic Anoxic Events, and Seafloor Hydrothermal Activity in the Jurassic and Cretaceous. *American Jounral of Science* **301**, 112–149 (2001).

46. Montero-Serrano, J. C. *et al.* Continental weathering and redox conditions during the early Toarcian Oceanic Anoxic Event in the northwestern Tethys: Insight from the Posidonia Shale section in the Swiss Jura Mountains. *Palaeogeogr Palaeoclimatol Palaeoecol* **429**, 83–99 (2015).
