# Supplementary material for: Global ocean redox changes before and during the Toarcian Oceanic Anoxic Event
Source: Nat Commun. 2023 Feb 13;14:815. doi: 10.1038/s41467-023-36516-x (PMC9925726; doi:10.1038/s41467-023-36516-x)
Supplement: Supplementary file 3 — Description of Additional Supplementary Files [file 41467_2023_36516_MOESM3_ESM.pdf]

Description of Additional Supplementary Files:

Supplementary Dataset 1: Elemental concentrations and organic carbon isotope compositions (δ 13Corg) for Toarcian samples. Model intervals (MI) are listed numerically from 1 to 4 up-section as in the main text. Redox interpretations are based on trace metal proxies as discussed in the Article. Depth is presented in meters (m) below surface. Isotopic compositions are given in per mil (‰). Organic carbon (Corg) and aluminium (Al) are given in weight percent (wt%), vanadium (V), manganese (Mn), cobalt (Co), molybdenum (Mo), cadmium (Cd) and uranium (U) in micrograms per gram (µg g-1 , i.e., parts-per-million) and rhenium (Re) in nanograms per gram (ng g-1 , i.e., parts-per-billion). Analytical techniques are described in Article Methods
